# Sensitivity and Specificity Analysis of Different Lesion Areas of Vitiligo by Reflectance Confocal Microscopy

**DOI:** 10.1111/jocd.70006

**Published:** 2025-02-07

**Authors:** Jiyuan Wu, Miaomiao Sun, Zijia Wei, Qian Jiang, Hongying Chen, Liuqing Chen

**Affiliations:** ^1^ Department of Dermatology Wuhan No. 1 Hospital Wuhan China; ^2^ Hubei Province and key Laboratory of Skin Infection and Immunity Wuhan China; ^3^ Hubei University of Chinese Medicine Wuhan China

**Keywords:** diagnosis, reflective confocal microscopy, refractory vitiligo, vitiligo, vitiligo staging

## Abstract

**Background:**

Clinical diagnosis and staging of vitiligo often rely on the patient's subjective recollection, physician's experience, or clinical evaluation methods. An objective, quantitative diagnostic approach is lacking.

**Aims:**

This study was performed to compare the sensitivity and specificity of different lesion areas and full scanning of lesion areas using reflectance confocal microscopy (RCM) for diagnosis and staging of vitiligo.

**Methods:**

Clinical and RCM data of patients diagnosed with vitiligo were collected. RCM data were gathered from three sites of a lesion: the center of the lesion, the margin of the lesion, and the perilesional normal skin. Scoring was conducted for each method, categorizing the results as either the progressive or stable stage of vitiligo.

**Results:**

The sensitivity was significantly lower in the perilesional normal skin than at multiple sites (*p* < 0.001). The specificity was significantly lower in the center of the lesion and at the margin than at multiple sites and significantly higher in the perilesional normal skin than at multiple sites (*p* < 0.05 for all).

**Conclusions:**

The results of RCM of different lesion areas of vitiligo showed that the rate of missed diagnosis of the progressive stage was lowest in the center of the lesion and comparable to that of multiple sites. The misdiagnosis rate for the stable stage was lowest in the perilesional normal skin, significantly differing from the other sites. Overall, scanning multiple sites yielded the most accurate results, offering flexibility in selection according to the patient's compliance.

## 1 Introduction

Vitiligo is a common skin disease characterized by acquired pigment loss. With advances in technology, vitiligo can now be distinguished from other hypopigmented skin conditions. Although clinical diagnosis is often straightforward, treatment can be difficult, impacting patients' social lives, and mental well‐being. Therefore, early diagnosis and accurate staging are crucial. Treatment strategies vary depending on the stage of vitiligo. Surgical interventions are typically preferred for patients in the stable stage, whereas medical therapy is commonly used for those in the progressive stage [1]. The primary focus of vitiligo treatment is addressing its underlying cause. This typically involves targeting the inflammatory response, enhancing melanocyte number and function, and promoting melanin redeposition [2, 3]. Treatment approaches vary according to the stage of vitiligo. In the stable stage, first‐line treatments mainly consist of narrow‐band ultraviolet B therapy, topical or systemic corticosteroids, and calcium‐modulated phosphatase inhibitors [4]. For patients experiencing rapid progression, oral small‐pulse therapy with steroids can be administered [5, 6].

Early and accurate determination of the stage of vitiligo can facilitate more effective treatment in a timely manner. For stable patients, this may involve prompt initiation of phototherapy or surgery. For patients experiencing progressive disease, the treatment strategy should focus on stopping the disease activity and promoting repigmentation. Reflective confocal microscopy (RCM), also known as skin computed tomography [7], can be used to diagnose dermatological diseases in real time, dynamically, and without the need for invasive procedures. By minimizing unnecessary biopsies, this approach reduces scarring and thus relieves patients' anxiety [8]. The results of RCM are more accurate and precise than those of previously used methods.

Although more precise staging of vitiligo is necessary, no uniform standard for staging vitiligo has yet been established in domestic and international guidelines [9]. RCM can be used as an adjunct to the diagnosis and treatment of pigmented skin diseases, and increasingly more studies are confirming that it can be used for the differential diagnosis, treatment, and prognosis of vitiligo [10, 11]. We found that the use of RCM to examine vitiligo lesions in different areas produces different results, and these differences have a certain impact on patient outcomes. To date, no study has focused on the use of RCM for different sites within vitiligo lesions.

## 2 Materials and Methods

### 2.1 General Information

This study involved 217 patients (121 male, 96 female) diagnosed with vitiligo at our dermatology outpatient clinic from March 2022 to December 2023. Clinical and RCM data of patients diagnosed with vitiligo were collected in accordance with standard reference guidelines for clinical data collection. The RCM data gathered from three lesion sites: site 1, lesional center; site 2, marginal; and site 3, perilesional normal skin. The image data primarily focused on the integrity of the pigment ring, boundary clarity, inflammatory infiltration, and dendritic melanocytes, all of which were scored using the skin computed tomography scoring method. The RCM characteristics were summarized and divided into four assessment methods: the center of the lesion, the margin of the lesion, the perilesional normal skin, and a multi‐site overall assessment. These four methods were scored, with the results classified into ‘progressive’ and ‘stable’ periods. The sensitivity and specificity of these four methods in determining the stage of vitiligo were then compared against clinical evaluation methods. The patients were aware of the diagnosis and treatment, were informed of the study purpose, and provided written informed consent. Because of the retrospective nature of the study, ethics approval was not required.

### 2.2 Inclusion Criteria

The study inclusion criteria were (1) a diagnosis of vitiligo according to the Diagnostic and Therapeutic Criteria for Melasma and Vitiligo (2010 Edition) [12] established by the Chinese Group of Integrative Medicine and Dermatological Pigmentology, (2) lesions of ≥ 2 cm in diameter, (3) age of ≤ 65 years, and (4) voluntary participation in the study with provision of written or electronic informed consent.

### 2.3 Exclusion Criteria

The study exclusion criteria were (1) poor compliance, missed visits, or the patient's desire to terminate treatment; (2) hormone therapy or phototherapy in the last month; (3) a history of severe cardiac, hepatic, renal, or neurological disease; (4) lesions in areas difficult to examine by RCM, such as the mucous membranes, lips, or mouth; (5) incomplete data collection that may affect the results of the examination; and (6) photosensitivity disease, a history of photosensitization, or allergies.

### 2.4 Clinical Evaluation Methods

Clinical evaluations were conducted by two specialized dermatologists. Patients with vitiligo who first visited our dermatology outpatient clinic were staged, and those whose diagnosis and staging of vitiligo were deemed consistent by both physicians were included in the study. The specific staging methods used were as follows.

### 2.5 Progressive Stage Determination

The following four criteria were used to assess the progressive stage of vitiligo, which included both rapid and slow progression. Patients were required to meet at least one of the four criteria.

- *VIDA score*: A score of +4, +3, +2, and + 1 indicated new or enlarged lesions in the last 6 weeks, 3, 6, and 12 months, respectively. The presence of new or enlarged lesions in the last 12 months was scored +1, whereas spontaneous pigment regeneration throughout at least 12 months was scored −1. A total VIDA score of > 1 indicated progression, ≥ 1 indicated advancement, and ≥ 4 indicated rapid progression.
- *Lesion characteristics*: Blurred edges, signs of inflammation (such as itching or erythema), trichrome vitiligo, and confetti‐like lesions or depigmented areas were indicative of progressive vitiligo.
- *Koebnerisation*: Koebnerisation was defined as the appearance of lesions in areas of skin injury within 1 year. Such skin injury may be physical (e.g., trauma, cuts, or scratches) or result from mechanical friction, persistent compression, thermal burns, cold injury, chemical exposure, allergic reactions (e.g., allergic contact dermatitis) or other inflammatory skin diseases, irritation (e.g., vaccination or tattooing), or treatments (e.g., radiation or phototherapy).
- *Wood's lamp examination*: Grayish‐white skin lesions with unclear borders and a larger area under the Wood's lamp than under visual inspection were suggestive of progression [13, 14].

### 2.6 Stable Stage Determination

The following four criteria were used to assess the stable stage of vitiligo. Patients were required to meet at least two of the four criteria.

- VIDA score of 0.
- *Clinical features*: Lesions exhibiting porcelain‐white coloration with well‐defined edges or pigmentation.
- Absence of Koënerisation for ≥ 1 year.
- *Wood's lamp examination*: Lesions appear white in color and have clear borders, and the lesion area is equal to or smaller than that under visual inspection with the Wood's lamp [13].

### 2.7 RCM Scoring Method

The skin lesions were selected to avoid mucous membranes and other areas unsuitable for scanning with the RCM instrument. All selected skin lesions were new‐onset leucoplakia with an area of ≥ 2 cm. RCM scanning was performed at three sites: the lesional center (site 1, the center of the lesion), the lesion margin (site 2, 0.75 cm within the boundary of the junction between the lesion and the normal skin), and the perilesional normal skin (site 3, 0.75 cm outside the boundary). The combined score of these three sites is hereinafter referred to as the ‘multi‐score’.

In the RCM scoring method, scores are assigned based on specific criteria. One point is added for each of the following conditions:

- *Incomplete loss of pigment ring*: RCM shows vitiligo lesions with incomplete loss of pigment ring integrity at the epidermal–dermal junction.
- *Unclear border*: RCM shows vitiligo lesions with obvious loss of the pigment ring and unclear borders with the surrounding normal skin.
- *Presence of inflammatory cells*: RCM shows highly refractive cells at the edge of the epidermal–dermal junction in vitiligo lesions.

By contrast, 1 point is subtracted for each of the following conditions:

- *Complete absence of pigment ring*: RCM shows vitiligo lesions with complete absence of the pigment ring.
- *Clear border*: RCM shows vitiligo lesions with complete absence of the pigment ring and clear borders with the normal skin.
- *Presence of dendritic melanocytes*: RCM shows dendritic, highly refractive melanocytes within vitiligo lesions.

Using this scoring system, a final total score of > 2, 1–2, and < 1 point indicates the rapidly progressive stage, slowly progressive stage, and stable stage, respectively [15, 16]. In multi‐site RCM examination, the scoring process begins by applying the single‐site scoring method to each individual site. The overall score for the multi‐site examination is then calculated as the sum of the scores of all three sites (sites 1, 2, and 3). A final total score of > 6, 3–6, and < 3 points indicates rapid progression, slow progression, and the stable stage, respectively.

### 2.8 Statistical Methods

Multiple independent samples were selected for the chi‐square test, and measures are expressed as mean ± standard deviation. Because there were no expected frequencies of < 5 in this study, the chi‐square test could be directly applied. The Kappa consistency test was used to assess the agreement between the multi‐site approach and the clinical evaluation methods, with analysis conducted using linearly weighted Kappa. The sensitivity and specificity of the RCM method compared with those of the clinical diagnosis method for both single scores and the multi‐score were evaluated using a dichotomous method and expressed as percentages. Statistical significance was determined using the chi‐square test, with the test level set at *α* = 0.05. A *p* value of < 0.05 indicated a statistically significant difference. Multiple comparisons by chi‐square testing were made with Bonferroni correction. All data were statistically processed using SPSS 27.0 (IBM Corp., Armonk, NY, USA).

### 2.9 Chi‐Square Tests Between Different Scoring Methods

With no expected frequencies of < 5, the chi‐square test could be directly used to compare the different scoring methods without correction.

A chi‐square test was performed to compare scanning a single lesion site versus multiple lesion sites. At a significance level of *α* = 0.05, the analysis showed a *p* value of < 0.001, indicating a statistically significant difference in the results of RCM scanning across different lesion sites. Multiple comparisons by chi‐square testing were made with Bonferroni correction, which indicated no significant difference between sites 1 and 2 when staging.

### 2.10 Consistency Test Between Multi‐Site RCM and Clinical Evaluation Criteria

The Kappa consistency test was used to compare the sum of previous scores with the multi‐site RCM score with weighting for the number of cases. The probability of significance of the Kappa test was *p* < 0.001, indicating consistency between the two tests. With a calculated Kappa value of 0.749 (slightly below 0.750) and an asymptotic standard error of 0.049, the results suggested that the scanning results of multi‐site RCM exhibited better agreement with the previous criteria, demonstrating consistency.

### 2.11 Comparison of Sensitivity and Specificity of Multi‐Site RCM With Clinical Evaluation Criteria

A dichotomous classification was used to categorize vitiligo into progressive and stable stages. The results of comparing the sensitivity and specificity of single‐site versus multiple‐site scanning based on previous criteria are shown below.

McNemar's test was used to compare the differences in sensitivity and specificity between the single‐site and multi‐site approaches. The results showed that compared with the findings using the multi‐site approach, the sensitivity of site 3 (perilesional normal skin) was significantly lower (*p* < 0.001), the specificity of sites 1 (lesional center) and 2 (margin) was significantly lower (*p* < 0.05), and the specificity of site 3 (perilesional normal skin) was significantly higher (*p* < 0.05). The areas under the curve were 0.726, 0.718, 0.770, and 0.862, indicating that the scanning results of multi‐site RCM were the most accurate.

## 3 Results

**FIGURE 1:**
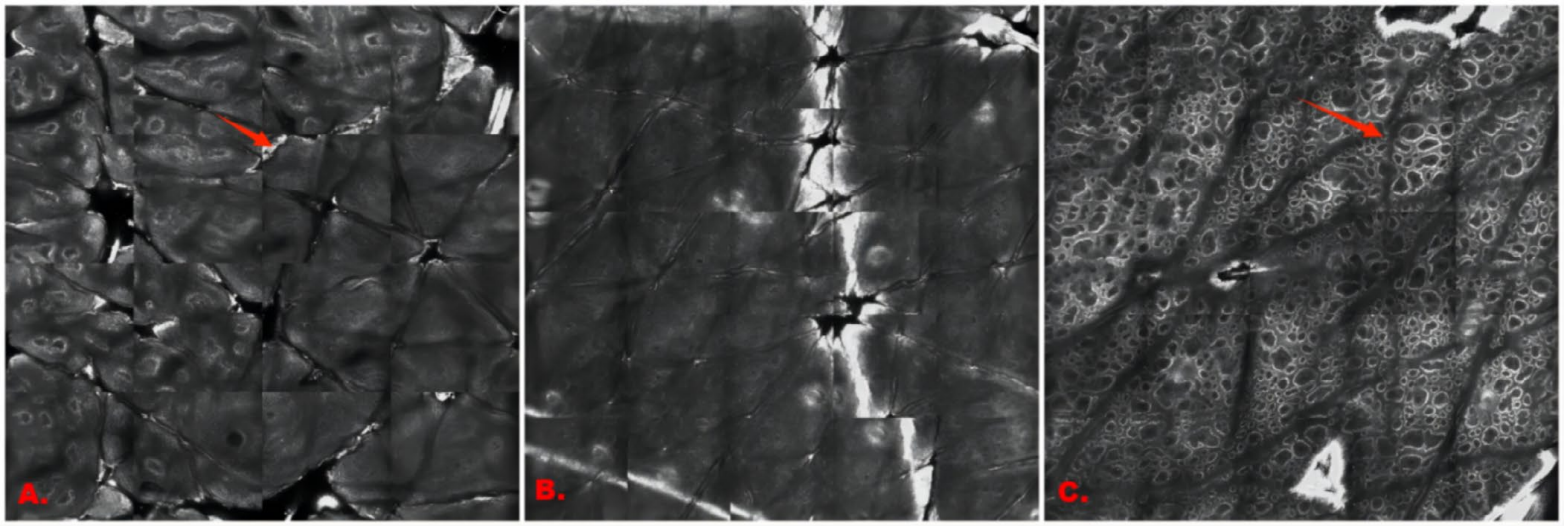
RCM images of different sites within a stable vitiligo lesion. (A) Marginal area: Clear border (red arrow). (B) Lesional area (center): Complete absence of the pigment ring. (C) Perilesional normal skin: Full pigment ring (red arrow), no sign of inflammatory cells.

**FIGURE 2:**
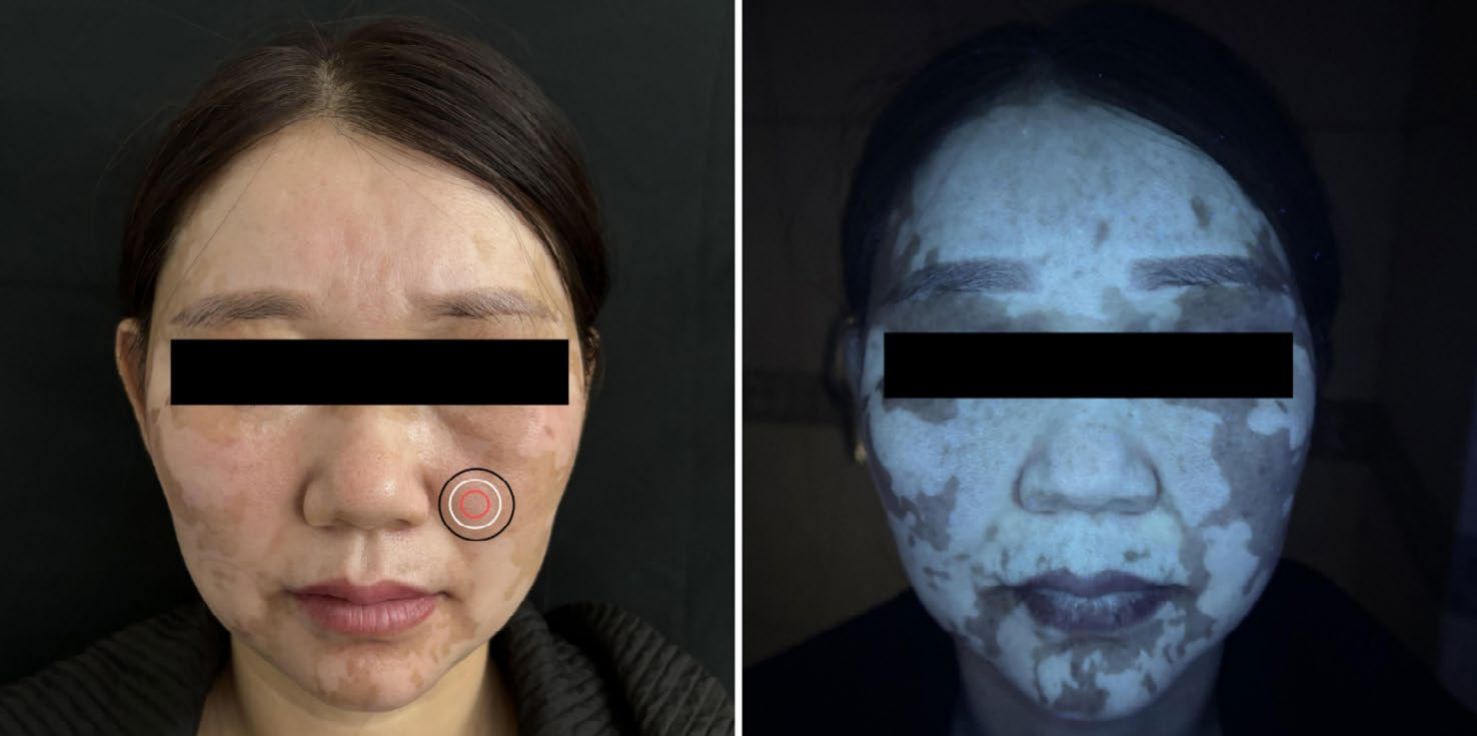
Clinical images of different sites within a stable vitiligo lesion. The red circle shows the lesion center (site 1), the white circle shows the lesion margin (site 2), and the black circle shows the perilesional normal skin (site 3).

As shown in Figure 1, the patient with vitiligo was clinically diagnosed as being in the stable stage. Images at the lesion margin showed clearly visible borders between the junction of the vitiligo lesion and the perilesional normal skin, with minor inflammatory cell infiltration and partial loss of the pigment ring's integrity. In the lesion center, the pigment ring was almost completely absent, with no significant inflammatory cell infiltration. In the perilesional normal skin, the pigment ring of the normal skin outside the vitiligo lesion was intact with no obvious pigment loss or significant inflammatory cell infiltration. No highly refractive dendritic melanocytes were observed in any of the three images. According to the RCM scoring method (single‐site: scores of ≥ 1 indicate progressive stage and < 1 indicate stable stage; multiple sites: multi‐score of ≥ 3 indicates progressive stage and < 3 indicate stable stage), the RCM scores were as follows: A: +1, B: −3, and C: −2. The RCM results for the lesion center and perilesional normal skin were in the stable stage, consistent with the clinical diagnosis. However, the marginal area showed ‘progressive stage (slow)’, which was inconsistent with the clinical diagnosis. The multi‐score was −4, aligning with the stable stage and consistent with the clinical diagnosis (Figure 2).

**FIGURE 3:**
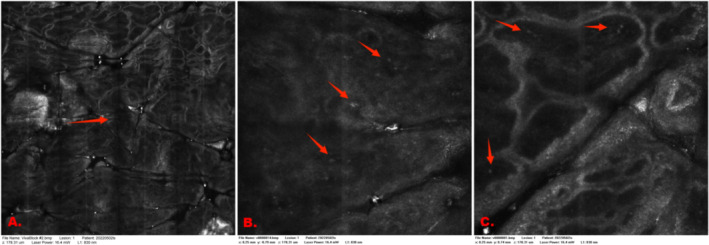
RCM images of different sites within a progressive vitiligo lesion. (A) Marginal area: Unclear border (red arrow). (B) Lesional area (center): Inflammatory cells (red arrow), loss of the pigment ring. (C) Perilesional normal skin: Refractive inflammatory cells (red arrow), rough basal cell ring.

**FIGURE 4:**
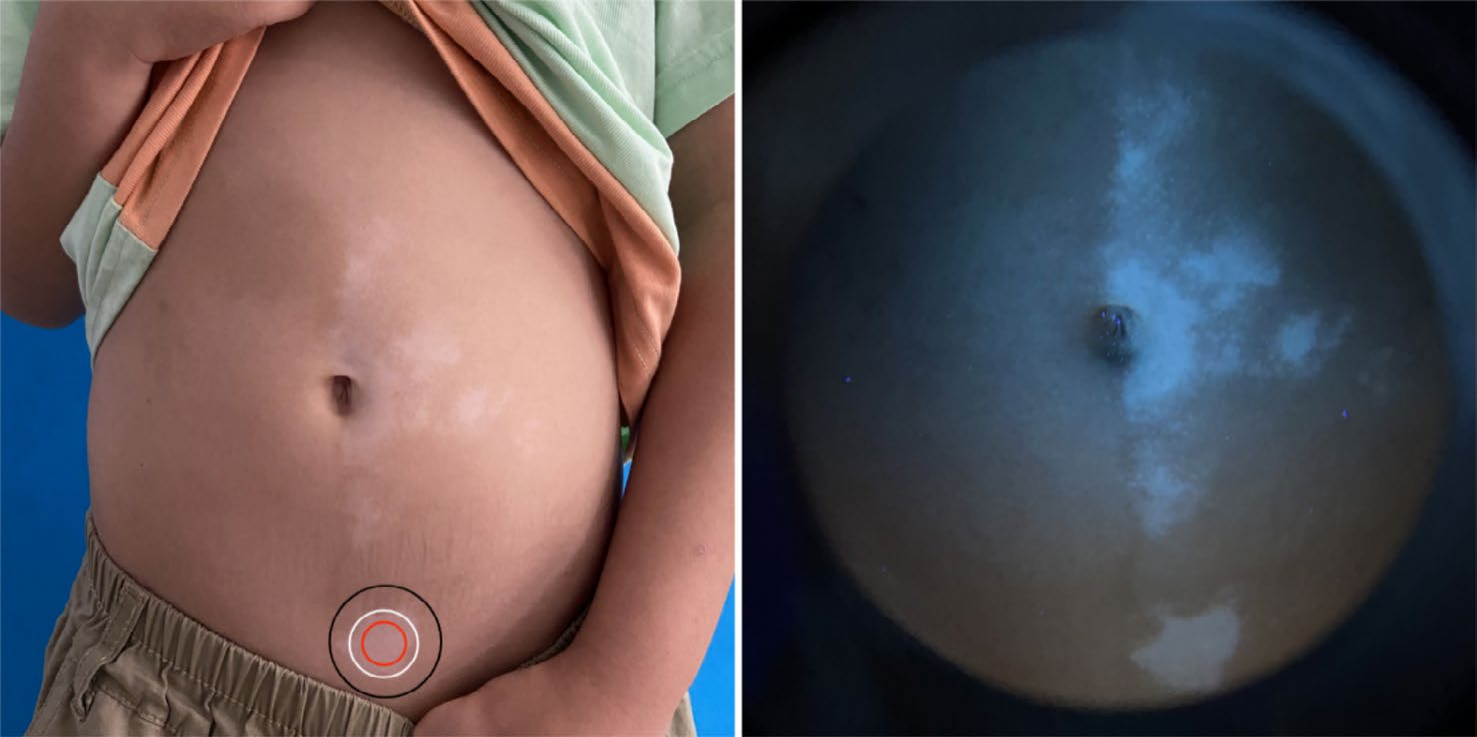
Clinical images of different sites within a progressive vitiligo lesion. The red circle shows the lesional center (site 1), the white circle shows the marginal (site 2), and the black circle shows the perilesional normal skin (site 3).

As shown in Figure 3, the patient with vitiligo was clinically diagnosed as being in the progressive stage. The image of the lesion margin showed an unclear boundary between the vitiligo lesion and perilesional normal skin, with an incomplete pigment ring and no significant inflammatory cell infiltration. In the center of the vitiligo lesion, the pigment ring was also incomplete, with a faint residual ring and highly refractive inflammatory cells. In the perilesional normal skin, the pigment ring was partially lost, with residual pigment and highly refractive inflammatory cells visible. No highly refractive dendritic melanocytes were observed in any of the three images. According to the RCM scoring method, the scores were A: +2, B: +3, and C: +3. The three RCM results indicated a ‘progressive stage’, consistent with the clinical diagnosis (but with different scores). The multi‐score was +8, which corresponded to the progressive stage and was consistent with the clinical diagnosis (Figure 4).

Following the RCM image analysis method described above, it is evident that RCM imaging results vary across different lesion sites. The staging results for images of single lesion areas and multiple areas are shown in Tables 1 and 2, while the statistical analysis of sensitivity and specificity is presented in Table 3, with the results summarized as follows.

**TABLE 1:** Chi‐square tests between different scoring methods.

Group	Cases with stable disease	Cases with progressive disease	Total
Site 1	36	181	217
Site 2	35	182	217
Site 3	116	101*,**	217
Multiple sites	61	156*,**,***	217
*p*‐value	*p* < 0.001		

* Statistically significant difference compared with site 1 (*p* < 0.05).

** Statistically significant difference compared with site 2 (*p* < 0.05).

*** Statistically significant difference compared with site 3 (*p* < 0.05).

**TABLE 2:** Consistency test between multi‐site RCM and clinical evaluation criteria.

		Clinical evaluation	
		Cases with stable disease	Cases with progressive disease
RCM Multi‐score	Cases with stable disease	54	7
	Cases with progressive disease	16	140
*p*‐value		*p* < 0.001	
Kappa consistency		0.749	

Abbreviation: RCM, reflectance confocal microscopy.

**TABLE 3:** Comparison of sensitivity and specificity of multi‐site RCM.

	Sensitivity	P _vs multi‐site_	Specificity	P _vs multi‐site_
Site 1	97.96	0.125	47.14	< 0.001
Site 2	97.96	0.125	45.71	< 0.001
Site 3	63.95	< 0.001	90.00	0.035
Multiple sites	95.24		77.14	

Abbreviation: RCM, reflectance confocal microscopy.

The above analyses show that the results of RCM at multiple sites were in good agreement with previous standards. In the assessment of the progressive stage of vitiligo, the sensitivity of sites 1 and 2 was the highest and the sensitivity of site 3 was the lowest, with a statistically significant difference. Conversely, in the assessment of the stable stage of vitiligo, the specificity of site 3 was the highest and the specificity of site 2 was the lowest, also with a statistically significant difference. Specifically, sites 1 and 2 exhibited high sensitivity (0.9796) in identifying the progressive stage of vitiligo, and the difference was not statistically significant when compared with multiple sites (*p* = 0.125). This suggests that scanning in the center of the lesion showed good sensitivity in detecting the progressive stage of vitiligo, and the rate of misdiagnosis was lower in patients with progressive vitiligo. Therefore, clinicians may consider performing multiple‐site scanning on a case‐by‐case basis according to both their experience and the patient's compliance (Table 4).

**TABLE 4:** Area under receiver operating characteristic curve.

	Area under the curve	Standard error	95% CI
Site 1	0.726	0.041	(0.645, 0.806)
Site 2	0.718	0.041	(0.637, 0.799)
Site 3	0.770	0.033	(0.705, 0.834)
Multiple sites	0.862	0.032	(0.800, 0.924)

Abbreviation: CI, confidence interval.

Site 3 had the highest specificity (0.90) in identifying stable vitiligo, with a statistically significant difference (*p* < 0.05). This indicates a lower misdiagnosis rate when RCM is performed in patients in the stable stage of vitiligo. Consequently, in clinical practice, scanning at the perilesional normal skin may be recommended for diagnosing patients in stable stages, patients with refractory vitiligo, and patients with ambiguous complaints.

## 4 Discussion

The results of this study highlight differences in the scanning results among various sites of vitiligo lesions under RCM. Although Liu and Xu [16] did not clearly propose the correlation between different stages and lesion sites in RCM‐assisted diagnosis of vitiligo, the selection of different lesion sites for diagnosis and treatment using RCM can facilitate more accurate staging determination. This optimized approach can enhance diagnosis and treatment planning, thereby preventing delays in managing the condition, especially for stable and refractory cases that do not readily undergo repigmentation.

RCM can provide imaging support for diagnosing, monitoring the progression, and assessing the prognosis of vitiligo. Its reproducibility allows for monitoring disease changes and treatment responses over time. Additionally, some studies have shown that pigmentation in vitiligo lesions can be induced by stem cell mobilization through melanocytes [17]. The appearance of melanocytes during vitiligo treatment, observed as pigmentation of lesions, can be observed under RCM. This capability is valuable for investigating the pathogenesis, repigmentation mechanisms, and pigmentation treatments of vitiligo [18].

RCM is among the most promising means of skin imaging in recent years. It offers several advantages, including its dynamic, noninvasive, and real‐time capabilities. RCM can produce three‐dimensional images of the epidermis and superficial dermis at the cellular level, aligning with pathological examination findings, and this capability is of great significance for the screening of dermatological diseases [19]. RCM has a wide range of applications in pigmented dermatoses, including its ability to examine vitiligo lesions at multiple sites within the lesions. This provides more accurate and quantitative results for the diagnosis and staging of vitiligo, enhancing clinicians' understanding of the specific conditions of referred patients. Unlike existing clinical evaluation methods that rely on patients' subjective and recollective complaints, RCM offers objective and real‐time insights.

In this study, we reviewed the recent literature on the use of RCM in the diagnosis of vitiligo both domestically and internationally. We then selected patients diagnosed with vitiligo using RCM in our department. Drawing upon the clinical experience of our department in the use of RCM technology, we examined the RCM data of patients either newly diagnosed or referred to us. We focused on the characteristics of vitiligo as depicted in the RCM images, specifically examining three sites: lesion center, lesion margin, and perilesional normal skin. Additionally, we explored the differences in results observed by RCM scanning of vitiligo lesions at different sites and assessed the advantages of multi‐site scanning.

The results of the study showed that the lesional center had good sensitivity in identifying the progression of vitiligo. Patients with progressive vitiligo can undergo examinations in selected sites within vitiligo lesions with a low rate of missed diagnosis. Additionally, patients with progressive vitiligo can be scanned at more than one site when needed or requested by the clinician. Such decision‐making is done on a case‐by‐case basis. Furthermore, perilesional normal skin showed the highest specificity (0.90) in determining stable vitiligo in this study, resulting in a low misdiagnosis rate when RCM is performed on stable patients. In clinical practice, scanning the perilesional normal skin is recommended for patients with stable and refractory vitiligo who exhibit poor outcomes.

The treatment of vitiligo, especially refractory vitiligo, remains challenging because the pathogenesis of vitiligo has not been fully elucidated. Examining different sites of vitiligo lesions by RCM offers insights into the diagnosis and treatment of vitiligo from a microscopic perspective, and melanin storage and recovery of difficult‐to‐repigment areas of vitiligo can be observed from a cellular perspective. This provides a certain degree of specificity when examining the lesions of patients with stable and refractory vitiligo. Microscopic examination helps to elucidate the reasons for the difficulty in repigmentation of vitiligo lesions. Additionally, assessing the integrity of the pigment ring and the number of dendritic cells using RCM provides valuable prognostic information. Further investigation with a larger sample size is warranted to confirm the effectiveness of repigmentation treatments. In progressive vitiligo, inflammatory infiltration is observed in the center of the lesion and at the margin, with higher sensitivity in the center of the lesion. The presence of inflammatory infiltration within lesions serves as a guiding factor for the prognosis. Moreover, the extent of inflammatory infiltration is correlated with disease progression, with more infiltration indicating faster progression of the disease. In such cases, treatment options may include oral minipulse therapy [20].

This study included 217 patients with vitiligo who were seen in our department from March 2022 to December 2023. The small sample size may have decreased the precision of our results. During the data analysis, we found that the consistency of the RCM scanning results for different lesion sites, including whole‐lesion scanning, significantly differed from the clinical evaluation method. Consistency at single sites was found to be moderate, aligning with findings reported by Guo et al. [21]. This inconsistency may stem from our limited sample size and the RCM scoring method used, which was based on existing RCM atlases and studies in China. In our scoring system, we made a careful distinction between the degree of pigment ring loss and the degree of inflammatory infiltration, even scoring tiny pigment ring losses and individual inflammatory cells. Additionally, the microscopic manifestations observed by RCM sometimes differed from those grossly observed in skin lesions, contributing to discrepancies with the clinical evaluation methods.

The application of this study also presents certain challenges that need addressing. From an objective point of view, the high cost of RCM limits its widespread use in clinical practice. Additionally, progressive vitiligo often manifests in multiple body areas, necessitating the clinician's judgment in selecting multiple lesion areas for examination. In the present study, the selection of vitiligo lesions was based on the most recent area of occurrence, which may have introduced some error into the results. Furthermore, there are inherent difficulties in the treatment of vitiligo, particularly refractory vitiligo that develops in special areas such as the mucous membranes, thin and tender areas, and extremities [22]. The vitiligo lesions characteristic of refractory vitiligo pose a challenge in RCM scanning, limiting detection capabilities. Thus, the exclusion of vitiligo lesions in special areas in this study may have also impacted the accuracy of the results. Subjectively, multi‐site examination of vitiligo progression and repigmentation requires a high degree of communication and cooperation between the doctor and patient, and the results of RCM are dependent on the doctor's experience and knowledge. Doctors must carefully select examination areas for multiple vitiligo lesions, considering the limitations of RCM scanning in certain areas. The dichotomous method employed in this study can only compare the sensitivity and specificity between the progressive and stable stages. It does not allow for further comparison of the sensitivity and specificity between the rapidly progressive, slowly progressive, and stable stages, which requires combined use of the previous criteria for diagnosis and treatment. Thus, the staging approach in this study has certain limitations and shortcomings. Although RCM scanning of different lesion sites has a certain degree of sensitivity and specificity and aligns well with clinical evaluation methods, it cannot replace clinical evaluation entirely in the diagnosis of vitiligo. Instead, it is necessary to engage in thorough communication with patients, gather a detailed medical history, and combine the RCM findings with the clinical diagnosis.

## Author Contributions

All authors have read and approved the final manuscript. W.J., S.M., and W.Z. performed the research. W.J., S.M., and W.Z. designed the study. C.L., J.Q. and C.H. contributed essential reagents or tools. W.J. and S.M. analyzed the data. W.J. and S.M. wrote the paper.

## Ethics Statement

The authors confirm that the ethical policies of the journal, as noted on the journal's author guidelines page, have been adhered to and the appropriate ethical review committee approval has been received. The Medical Ethical Committee of the Wuhan No. 1 Hospital (MEC‐2024‐82).

## Conflicts of Interest

The authors declare no conflicts of interest.

## Data Availability

Research data are not shared.
